# Dr. Henry D. Clapp

**Published:** 1908-05

**Authors:** 


Dr. Henry D. Clapp, of Geneva, N. Y., died at his home in that city March 19, 1908, from pulmonary edema after a prolonged illness, aged 34 years. He graduated at the University of Buffalo, medical department, in 1895, and afterward served as interne at the Rochester City Hospital.

